# General Offending and Intimate Partner Violence Perpetration in Young Adulthood: A Dutch Longitudinal Study

**DOI:** 10.1177/0306624X211022657

**Published:** 2021-06-06

**Authors:** Janna Verbruggen, Arjan A. J. Blokland, Amanda L. Robinson, Christopher D. Maxwell

**Affiliations:** 1 Vrije Universiteit Amsterdam, The Netherlands; 2 Leiden University, The Netherlands; 3 Cardiff University, UK; 4 Michigan State University, East Lansing, USA

**Keywords:** intimate partner violence, general offending, young adulthood

## Abstract

This study examines the relationship between general offending and intimate partner violence (IPV) perpetration in young adulthood, using a Dutch longitudinal study. Young adults were followed over four waves, and self-reported data on general offending, IPV perpetration, and a number of individual characteristics were collected. Results of random effects models demonstrated that young adults involved in more diverse offending behavior reported higher levels of different types of IPV perpetration, even when individual factors were taken into account. Moreover, logistic regression analyses showed that general offending was also related to an increased likelihood of continuity in IPV perpetration. Taken together, the findings indicate that it is useful to view IPV perpetration as part of a broader criminal career.

## Introduction

Intimate partner violence (IPV), which can consist of a range of behaviors including physical aggression, psychological abuse, and sexual coercion, is a serious social problem (World Health Organization, 2017). Research conducted in the EU countries has shown that on average 20% of women have suffered from physical IPV, 43% have reported psychological abuse, and 7% have experienced sexual IPV, since the age of 15 (European Union Agency for Fundamental Rights [FRA], 2014). Dutch research indicated that 6% of adults have experienced physical, sexual, and/or psychological IPV over the past 5 years, with significantly more women (8%) than men (5%) experiencing victimization (Van der Veen & Bogaerts, 2010). Moreover, prevalence rates of IPV perpetration and victimization are highest among young adults (Desmarais et al., 2012; FRA, 2014). Despite relatively high rates of IPV in young adulthood, longitudinal studies have also demonstrated that in general, rates of IPV perpetration tend to decrease throughout young adulthood (Johnson et al., 2015; Kim et al., 2008; Shortt et al., 2012). To illustrate, Johnson et al. (2015) studied the development of IPV perpetration from ages 13 to 28, and found that prevalence of IPV perpetration peaks around age 20, and then declines throughout young adulthood.

Thus, it appears that the development of IPV perpetration mirrors the development of general antisocial and offending behavior, which has also extensively been shown to be prevalent during the teenage years, but decrease throughout young adulthood (e.g., Gottfredson & Hirschi, 1990; Sweeten et al., 2013). However, thus far the field of IPV research has developed largely separate from life course criminological research on the development of general offending behavior. Research has only recently begun to examine IPV perpetration as part of a broader general criminal career, and this body of work points to an overlap between general offending and IPV perpetration (e.g., Capaldi et al., 2012; Piquero et al., 2006). Therefore, the aim of this paper is to add to this burgeoning area by examining the association between general offending and IPV perpetration in young adulthood.

## The Association between General Offending and IPV Perpetration

Traditionally, IPV-focused scholars have viewed this form of violence as largely unrelated to general offending, and have instead emphasized the importance of gender inequality and gender roles (Dobash & Dobash, 1979), or focused on family-related processes including power dynamics, family conflict, and stress (Straus et al., 1980). Subsequently, more integrated, multi-factor models for understanding IPV have emerged that recognize an association between general antisocial or violent behavior and IPV perpetration. In these models, general antisocial behavior is identified as a risk factor for IPV perpetration, along with other factors, such as relationship dynamics, a partner’s traditional gender ideology, and other proximal triggers and stressors (Bell & Naugle, 2008; Capaldi & Clark, 1998; Capaldi et al., 2005; DeMaris et al., 2003). Furthermore, researchers who have proposed typologies have indicated that a subgroup of IPV perpetrators who engage in generally violent and antisocial behavior in addition to IPV tend to engage in more serious and persistent IPV (Holtzworth-Munroe & Stuart, 1994, Holtzworth-Munroe et al., 2003).

A different perspective is offered by general theories of crime and violence, which argue that various forms of antisocial behavior stem from similar underlying risk factors or a certain criminal propensity. For instance, Gottfredson and Hirschi (1990) assume that individuals with low levels of self-control, a trait which is thought to form early in life and remain relatively stable over time, will engage in different types of antisocial and criminal behavior over the life course. Low self-control has been found to be associated with both general criminal behavior (Pratt & Cullen, 2000; Vazsonyi et al., 2017) and physical IPV perpetration (Payne et al., 2010; Sellers, 1999).

In addition, Moffitt (1993) states that a small group of individuals who develop a stable pattern of antisocial behavior early in life due to an interaction between early individual risk factors and a criminogenic social environment are at risk of engaging in a wide range of antisocial behaviors throughout their lives in different social settings. To illustrate, research examining a range of outcomes among young adults at age 26 showed that those involved in persistent serious antisocial behavior throughout childhood and adolescence showed higher rates of physical and controlling IPV perpetration in young adulthood compared to those whose level of antisocial behavior was normative (Moffitt et al., 2002). Furthermore, persistent offending has also been found to be associated with physical IPV perpetration beyond young adulthood (Piquero et al., 2014; Verbruggen et al., 2019).

Research has also demonstrated that experiencing child abuse and family violence in childhood is a key risk factor for subsequent antisocial and violent behavior, including both general criminal behavior and IPV in relationships (e.g., Ehrensaft et al., 2003; Gómez, 2011; Lansford et al., 2007; Milaniak & Widom, 2015; Sunday et al., 2011; see also Capaldi et al., 2012, Costa et al., 2015, and Murray & Farrington, 2010 for reviews), due to processes of social learning (Mihalic & Elliott, 1997; Widom, 1989). Indeed, research using a nationally representative sample of youths found that a history of child abuse was predictive of IPV perpetration in young adulthood (Gómez, 2011). Furthermore, Milaniak and Widom (2015) found that young adults who experienced child abuse were more likely than those who had not to engage in criminal violence and physical IPV perpetration. Moreover, other research has demonstrated that youths whose parents had physically abused them were more likely than non-abused youths to subsequently be verbally and physically aggressive toward a partner (Sunday et al., 2011).

Although general theories of crime and violence do not always explicitly address IPV perpetration, different manifestations of antisocial behavior, including general offending and IPV perpetration, share key risk factors and to a certain extent appear to have a similar etiology (Fagan & Wexler, 1987; Felson & Lane, 2010; Moffitt et al., 2000). Following this, an association between general offending and IPV perpetration in young adulthood is expected. Indeed, a growing body of research has established an association between general crime and IPV perpetration, including self-report studies that demonstrate that involvement in antisocial behavior and crime, especially serious and persistent antisocial behavior, is a risk factor for later IPV perpetration in young adulthood (Capaldi & Clark, 1998; Ehrensaft et al., 2003; Herrenkohl et al., 2007; Kim & Capaldi, 2004; Magdol et al., 1998; Novak & Furman, 2016; Woodward et al., 2002), and research using officially registered data on judicial samples, which indicates that arrested IPV perpetrators often also commit other violent and non-violent offenses (Buzawa & Hirschel, 2008; Hilton & Eke, 2016; Klein & Tobin, 2008; Piquero et al., 2006), and that offenders who violently offend in public settings also commit violence in the privacy of their homes (Van Ham et al., 2017).

However, most existing longitudinal research on the association between general offending and IPV perpetration has been conducted in the US and other Anglo-Saxon countries. It is unknown to what extent these findings are generalizable to continental European countries, such as the Netherlands, which differs considerably from the US. For example, there are fewer socioeconomic risk factors for crime and violence in the Netherlands compared to the US, due to lower levels of poverty and inequality, and better welfare state support (Alber, 2006). Moreover, levels of gender equality in the Netherlands are relatively high. Although higher levels of gender equality have also been found to be associated with increased reporting of IPV, possibly due to increased awareness and recognition of the phenomenon, it is generally thought to result in lower actual levels of IPV (FRA, 2014). Indeed, estimates of the prevalence of IPV are lower in Western-European countries such as the Netherlands, compared to the US (World Health Organization, 2013), as are rates of violence generally (United Nations, 2016). As it is largely unknown whether these types of cross-national differences and generally lower rates of (intimate partner) violence impact the generalizability of research findings on the association between general offending and IPV perpetration, the current study aims to build upon existing research evidence by examining the association between general offending and IPV perpetration using a longitudinal dataset from the Netherlands.

Most existing research on the association between general offending and IPV perpetration focuses on physical IPV (but see, e.g., Kim & Capaldi, 2004), and longitudinal studies that include measures of other types of IPV, such as sexually abusive acts and psychological abuse, are limited (Costa et al., 2015). As research has shown that IPV often not only consists of physical abuse, but rather comprises manifold behaviors that serve to exert control over a partner (Stark, 2007), the current study includes psychological IPV and sexual coercion, along with physical IPV.

Using data from a longitudinal study of young adults in the Netherlands, the current study aims to examine, first, to what extent general offending is related to both different forms of IPV perpetration in young adulthood and the likelihood of continuity in IPV perpetration. Secondly, as general theories of crime and violence assume that stable, underlying factors contribute to both general offending and IPV, the study also aims to examine to what extent any observed association between general offending and IPV perpetration holds when stable background risk factors, including low self-control and history of family violence, are accounted for. It is expected that young adults who show higher levels of general offending are involved in higher rates of IPV perpetration, but that this association can be partly explained by stable individual characteristics.

## Methods

### Sample

This study uses data from the Transitions in Amsterdam (TransAM) study (Blokland, 2014), a Dutch longitudinal study conducted by the Netherlands Institute for the Study of Crime and Law Enforcement (NSCR). The primary aim of TransAM is to examine the relationships between criminal behavior and transitions in a variety of personal and life domains among young adults living in Amsterdam. Data collection was carried out in four waves between 2010 and 2014. For the first wave, 3,408 young adults across three age cohorts (18, 19.5, and 21 years old) were randomly selected via the municipal registry. Due to the study’s focus on criminal behavior during young adulthood, young adults with certain characteristics were oversampled, namely those who had contact with the police prior to age 17, and those from Dutch-Moroccan and Dutch-Antillean descent, as these groups are overrepresented in Dutch crime statistics (for more information about the study, see Hill, 2017).

Young adults were sent a letter with information about the study, after which trained interviewers visited them at their homes to invite them to participate in the study. Overall, 970 young people participated in wave 1 of the study (28.5% recruitment rate). Those that participated in wave 1 were invited to participate in three more waves, each 6 months apart (wave 2 *N* = 829; wave 3 *N* = 778; wave 4 *N* = 693). In waves 1 and 4, trained interviewers conducted face-to-face interviews with participants, using a structured interview schedule on a laptop. For waves 2 and 3, respondents participated in a shorter interview conducted by telephone, along with completing an online questionnaire. Questions on sensitive topics, including offending and IPV, were included in a self-report module on the laptop in waves 1 and 4, and in the online questionnaire rather than the telephone interview in waves 2 and 3. Participants received a voucher after completing the interviews.

Out of all the respondents, 555 young people (57.2%) reported being in a relationship during at least one of the waves. Given that the current study aims to examine the association between general offending and IPV perpetration throughout young adulthood, we focus on a subsample of young adults who reported being in a relationship, and therefore answered questions about IPV, in at least two of the four waves (*N* = 352).^1^

### Data and Measures

#### IPV perpetration

Information on IPV perpetration was collected using the Revised Conflict Tactics Scale (CTS2; Straus et al., 1996), which was translated into Dutch. For this study, three different scales were used. The psychological aggression scale comprised eight items, including “I shouted or yelled at my partner,” and “I threatened to hit or throw something at my partner.” The physical assault scale contained 12 items, such as “I slapped my partner,” and “I beat up my partner.” Sexual coercion was measured by seven items, including “I insisted on sex when my partner did not want to,” and “I used threats to make my partner have sex.” Reliability of the scales was good across the four waves (psychological aggression scale: Cronbach’s alphas .77 to .81, Guttman’s lambda-2 .80 to .83, physical assault scale: Cronbach’s alphas .81 to .87, Guttman’s lambda-2 .84 to .89, sexual coercion scale: Cronbach’s alphas .63 to .79, Guttman’s lambda-2 .73 to .84 (Guttman, 1945)).

Respondents reported how often they had engaged in these different acts during the past 6 months. Per item, these values were recoded into a frequency measure as follows: never (0), once (1), twice (2), 3 to 5 times (4), 6 to 10 times (8), 11 to 20 times (15), or more than 20 times (21). For each wave, all items were summed to create a total measure for frequency of *any IPV perpetration*. Items were also summed by scale to construct frequency measures of *psychological IPV, physical IPV*, and *sexual coercion* for each wave.

Furthermore, measures of continuity in IPV perpetration were constructed. Respondents were coded as showing *continuity in any IPV perpetration* if they had reported any of the IPV behaviors at least once in two or more waves. Measures for *continuity in psychological IPV*, *continuity in physical IPV*, and *continuity in sexual coercion* were similarly constructed.

#### General offending

In this study, we use the term *general offending* to denote all self-reported offending that is not IPV offending. General offending was measured in all four waves using a self-report delinquency scale comprising 48 items, based on items from the Self-Reported Delinquency study (Junger-Tas et al., 1994) and the South Holland study (Hofstra et al., 2001). Respondents were asked whether they had committed any of the 48 delinquent acts during the past 6 months (in wave 1) or since the previous interview (in waves 2–4). Respondents reported on a variety of delinquent acts, ranging from minor offenses like graffiti to serious offenses such as robbery. A total of 36 items measured non-violent offenses, including property offenses, damage to property, and drug-related offenses, while 12 items concerned violent offenses, such as threatening to use violence, hitting and/or kicking someone on purpose and injuring them, and using violence to steal from someone.^2^ Reliability of the self-reported offending scale was good across the waves (Cronbach’s alphas .71 to .87, Guttman’s lambda-2 .73 to .88).

The individual dichotomous items were summed to create a variety scale for *general offending.* The general offending variable hence represents the number of different offenses committed, a higher score on this general offending variable indicating more diverse offending. Variety scales of self-reported offending are recommended over dichotomous scales, which reduce variation among offenders, and frequency scales, which can comprise high frequency reporting of relatively minor acts of antisocial behavior (Sweeten, 2012).

#### Demographic and individual characteristics

Several demographic and individual characteristics measured at wave 1 were included in the analyses as control measures, as they could influence both general offending and IPV perpetration. Regarding demographic factors, a time-varying measure for age was included in the analyses, along with time-stable measures for gender (0 = female, 1 = male), ethnicity (0 = Dutch, 1 = Dutch-Antillean, 2 = Dutch-Moroccan),^3^ and educational level at wave 1 (1 = low, 2 = medium, 3 = high).

Furthermore, several individual characteristics have been identified in the literature as potentially important underlying explanatory mechanisms for both offending and IPV perpetration. Therefore, we included these measures, namely family violence in childhood and level of self-control, in the analyses, to examine to what extent general offending is related to IPV perpetration when these factors are accounted for.

*Family violence in childhood* was measured by a scale comprising six items, four of which measured witnessing violence between parents, or parents and siblings (i.e., father or mother hitting or kicking each other or siblings), and two items measured experiencing violence (i.e., father or mother hitting or kicking respondent). Respondents answered on a 5-point scale using the following options: never, once or twice, several times, every month, and every week. These answers were used to construct a dichotomous measure that captured whether respondents had witnessed and/or experienced one or more types of family violence several times or more (i.e., never, and once or twice = 0; several times, monthly, and weekly = 1). The scale was reliable in this study (Cronbach’s alpha = .87, Guttman’s lambda-2 = .87).

The Self-Control Scale (Grasmick et al., 1993) was used to measure respondents’ *level of self-control*. This questionnaire comprised 24 items, and included six subscales: impulsivity, simple tasks, risk seeking, physical activities, self-centered, and temper. Respondents indicated on a scale from 1 (strongly disagree) to 4 (strongly agree) to what extent they agreed with statements such as “I often act on the spur of the moment without stopping to think,” “I will take a risk just for the fun of it,” and “I lose my temper pretty easily.” The average score per respondent was calculated, with a higher score indicating higher levels of impulsivity, risk seeking, etc., and therefore *lower* levels of self-control (or higher levels of “antisocial propensity”). Reliability of the scale in this study was good (Cronbach’s alpha = .82, Guttman’s lambda-2 = .83).

### Analysis

First, descriptive statistics and bivariate analyses were used to describe the prevalence of IPV perpetration and general offending across the four waves. Second, random effects models were estimated to examine the association between general offending and different types of IPV perpetration, whilst taking relevant demographic and individual factors into account. Random effects models can be used to estimate between-individual differences (Andreß et al., 2013). For this study, this means that the models were used to examine whether throughout early adulthood, respondents reported significantly higher levels of IPV perpetration at times when they were also involved in more diverse general offending. Moreover, in addition to examining the relationship between general offending and IPV perpetration, the current study also aims to take the effects of time-stable independent measures into account, including the individual’s history of childhood family violence and low self-control. Random effects models allowed us to estimate the effects of these time-stable independent measures in addition to the effect of the time-varying independent measure for general offending on IPV perpetration (Andreß et al., 2013).

Four random effects models were estimated with different measures of IPV perpetration as dependent measures, namely any IPV perpetration (model 1), psychological IPV perpetration (model 2), physical IPV perpetration (model 3), and sexual coercion (model 4). As these outcome measures are counts, a negative binomial model was estimated. The general offending measure (i.e., the number of different offenses at each wave) was included as an independent measure in all models. Moreover, several factors that potentially influence the association between general offending and IPV perpetration were included in the models, namely age, gender, ethnicity, educational level, family violence in childhood, and low self-control.

Finally, four binary logistic regression analyses were conducted to examine the association between general offending (i.e., sum of the different types of offending reported in the different waves), demographic and individual factors, and the likelihood of continuity in any IPV perpetration (model 5), psychological IPV perpetration (model 6), physical IPV perpetration (model 7), and sexual coercion (model 8).

## Results

### Demographic and Individual Characteristics

The subsample used for the current study (*N* = 352) comprised 60.8% females (*N* = 214) and 39.2% males (*N* = 138). Over half the sample (53.1%; *N* = 187) were Dutch, a quarter were Dutch-Moroccan (*N* = 88), while 21.9% were of Dutch-Antillean descent (*N* = 77). Respondents were on average aged 20 at the start of the study (*M* = 20.12; *SD* = 1.31), aged 20.66 (*SD* = 1.32) at wave 2, aged 21.13 (*SD* = 1.30) at wave 3, and almost aged 22 at wave 4 (*M* = 21.76; *SD* = 1.30). The educational level of the majority of respondents was either medium (40.6%) or high (46.3%). Regarding individual characteristics, around one in five young adults (*N* = 67) had witnessed and/or experienced family violence in childhood several times or more. The average level of self-control was 2.27 (*SD* = 0.32) on a scale from 1 to 4.

### IPV Perpetration in Young Adulthood

Table 1 shows the proportion of respondents who reported IPV perpetration per wave. In wave 1, almost 80% of young people engaged in some form of IPV perpetration. This proportion was lower (65%–70%) in subsequent waves. However, these prevalence rates were driven by psychological IPV perpetration, which was relatively common across the waves. Regarding physical IPV perpetration, the results showed that in wave 1, over 40% of young adults reported physical IPV, while this decreased over the waves to 22% in wave 4. Approximately 10% of young adults reported sexual coercion in each wave.^4^

**Table 1.** IPV Perpetration by Wave.

	Wave 1 (*N* = 283)		Wave 2 (*N* = 275)		Wave 3 (*N* = 269)		Wave 4 (*N* = 245)	
Prevalence of IPV perpetration								
	*N*	%	*N*	%	*N*	%	*N*	%
Any IPV	224	79.2	191	69.5	176	65.4	164	66.9
Psychological IPV	219	77.4	188	68.4	171	63.6	163	66.5
Physical IPV	118	41.7	68	24.7	60	22.3	54	22.0
Sexual coercion	29	10.2	34	12.4	30	11.2	23	9.4
One type of IPV	106	37.5	116	42.2	110	40.9	102	41.6
Two types of IPV	94	33.2	51	18.5	47	17.5	48	19.6
Three types of IPV	24	8.5	24	8.7	19	7.1	14	5.7
Number of different types of IPV (among those involved in IPV)								
	*M*	*SD*	*M*	*SD*	*M*	*SD*	*M*	*SD*
Types of IPV	1.63	0.67	1.52	0.71	1.48	0.68	1.46	0.65
Frequency of IPV perpetration (total sample)								
Any IPV	17.61	32.36	12.76	26.38	10.79	25.17	10.50	21.95
Psychological IPV	12.78	20.05	8.31	14.79	7.49	14.42	7.64	14.21
Physical IPV	4.07	12.96	3.27	10.70	2.53	10.38	2.36	8.09
Sexual coercion	0.76	3.85	1.17	5.34	0.77	3.57	0.51	3.18
IPV perpetration in two or more waves (*N* = 352)	*N*	%						
Any IPV	255	72.4						
Psychological IPV	247	70.2						
Physical IPV	86	24.4						
Sexual coercion	25	7.1						

Furthermore, approximately 40% of young adults in each wave were involved in only one type of IPV perpetration. In wave 1, one in three respondents reported two types of IPV perpetration, but this decreased in subsequent waves to under 20%. A small proportion of young adults (6%–9%) reported all three types of IPV perpetration across the four waves. Moreover, among those who engaged in IPV perpetration, the average number of types of IPV reported decreased slightly over the waves from 1.63 (*SD* = 0.67) to 1.46 (*SD* = 0.65).

Regarding the frequency of the different types of IPV perpetration, the average number of abusive acts reported was highest for psychological IPV, and decreased across the waves for both psychological IPV (from *M* = 12.78 in wave 1 to *M* = 7.64 in wave 4) and physical IPV perpetration (from *M* = 4.07 in wave 1 to *M* = 2.36 in wave 4), whereas the average number of sexually coercive acts declined after wave 2.

Finally, Table 1 indicates a degree of continuity in IPV perpetration over the waves, albeit primarily regarding psychological IPV, with 70% of young adults reporting psychological IPV in two or more waves. The proportions of respondents displaying continuity in physical IPV and sexual coercion were lower, 24.4% and 7.1%, respectively.

### General Offending in Young Adulthood

Self-reported general offending rates across the four waves are displayed in Table 2. In wave 1, 36.7% of respondents reported at least one offense. The proportion of young adults involved in general offending decreased slightly across the waves, although an increase was observed in wave 4. Of those young adults who committed at least one offense, most reported non-violent offending, while a smaller proportion engaged in violent offending. The average number of different offenses reported fluctuated from wave to wave. Given that respondents could report up to 48 different offenses, the average number of different offenses was low (0.65–0.89 across the waves), although the average number of different offenses committed was considerably higher when only looking at those who reported at least one offense (2.11–2.69 across the waves).

**Table 2.** General Offending by Wave.

	Wave 1 (*N* = 349)		Wave 2 (*N* = 323)		Wave 3 (*N* = 310)		Wave 4 (*N* = 298)	
Prevalence of offending	*N*	%	*N*	%	*N*	%	*N*	%
Any offending	128	36.7	99	30.7	85	27.4	98	32.9
Non-violent offending	120	34.4	92	28.5	78	25.2	92	30.9
Violent offending	36	10.3	23	7.1	21	6.8	27	9.1
Number of different offenses (total sample)	*M*	*SD*	*M*	*SD*	*M*	*SD*	*M*	*SD*
Any offending	0.85	1.57	0.65	1.54	0.71	2.05	0.89	2.55
Non-violent offending	0.68	1.24	0.53	1.22	0.57	1.55	0.71	1.98
Violent offending	0.16	0.58	0.12	0.56	0.15	0.67	0.17	0.74
Number of different offenses (among offenders)								
Any offending	2.30	1.84	2.11	2.17	2.60	3.23	2.69	3.88
Non-violent offending	1.84	1.42	1.73	1.66	2.07	2.38	2.17	2.96
Violent offending	0.44	0.89	0.38	0.96	0.53	1.20	0.52	1.22

### The Association between General Offending, Demographic and Individual Characteristics, and IPV Perpetration

A series of random effects models were estimated to examine whether young adults who engaged in offending showed significantly higher levels of IPV perpetration. Moreover, the models examined whether any observed association held when controlling for relevant demographic and individual characteristics which may influence both the likelihood of general offending and IPV perpetration, including age, gender, ethnicity, educational level, family violence in childhood, and low self-control (Table 3).

**Table 3.** The Association between General Offending, Demographic and Individual Characteristics, and IPV Perpetration (*N* = 352).

	Model 1: Any IPV		Model 2: Psychological IPV		Model 3: Physical IPV		Model 4: Sexual coercion	
	B	*SE*	B	*SE*	B	S*E*	B	*SE*
General offending	0.07***	0.01	0.07***	0.01	0.09***	0.02	0.08**	0.03
Age	−0.08*	0.03	−0.09*	0.03	−0.19**	0.06	−0.02	0.07
Gender	0.02	0.10	0.01	0.11	0.03	0.18	0.80***	0.21
Dutch-Antillean	0.10	0.13	0.08	0.13	0.33	0.21	0.59*	0.27
Dutch-Moroccan	−0.27*	0.13	−0.29*	0.13	−0.09	0.21	1.01***	0.24
Education level	0.27***	0.08	0.31***	0.08	0.30*	0.14	0.06	0.17
Family violence	0.64***	0.12	0.64***	0.13	0.57**	0.20	−0.03	0.25
Low self-control	0.43*	0.17	0.31^†^	0.18	0.82**	0.29	1.19**	0.35
Constant	−0.39	0.80	0.20	0.84	−0.09	1.36	−6.35***	1.72

† *p* < .10. *p < .05. **p < .01. ***p < .001.

Model 1 examined the associations between general offending, demographic and individual factors, and any IPV perpetration. Results indicated that general offending was significantly associated with IPV perpetration, even when demographic and individual factors were taken into account, indicating that respondents showed increased levels of IPV perpetration in those waves in which they committed a higher number of different offenses. Moreover, family violence and lower levels of self-control were also associated with significantly higher rates of IPV perpetration. Regarding the demographic factors, older respondents showed lower levels of IPV, while higher educational levels were associated with increased IPV perpetration. Finally, Dutch-Moroccan respondents reported lower levels of IPV than Dutch respondents.

Models 2, 3, and 4 examined the association between general offending, demographic and individual factors, and different types of IPV perpetration. Regarding psychological (model 2) and physical (model 3) IPV perpetration, the results were largely similar to model 1. General offending was significantly related to psychological and physical IPV perpetration, insofar as when respondents reported a higher variety of committed offenses they also reported higher rates of psychological and physical IPV perpetration. These effects remained significant when controlling for family violence in childhood and lower levels of self-control, which were also significantly related to levels of psychological and physical IPV perpetration, albeit the effect of low self-control was marginally significant in model 2 (psychological IPV). Moreover, respondents with higher levels of education engaged in higher rates of psychological and physical IPV, whereas older respondents reported lower levels of psychological and physical IPV. Finally, young adults of Dutch-Moroccan descent reported lower rates of psychological IPV than Dutch respondents.

When examining the association between general offending and sexual coercion in model 4, findings again showed that when respondents committed a larger number of different general offenses, they reported significantly higher rates of sexually coercive IPV. Furthermore, males engaged in significantly higher rates of sexual coercive behavior than females, as did Dutch-Antillean and Dutch-Moroccan respondents compared to their Dutch counterparts. Low self-control was significantly associated with sexually coercive IPV, whilst family violence in childhood was not significantly related to the level of sexual coercion in relationships.

In sum, the results of the random effects models indicate that the higher the number of different offenses reported, the higher the rate of IPV perpetration. When the demographic and individual factors were added to the models, the effects of general offending became slightly smaller, but all remained significant (only full models are shown in Table 3), thus suggesting that, even when background factors are accounted for, an important association between criminal behavior and various forms of IPV perpetration exists.^5^

### The Association between General Offending, Demographic and Individual Characteristics, and Continuity in IPV Perpetration

Finally, a series of binary logistic regression analyses (models 5–8) were conducted to examine the association between general offending, demographic and individual characteristics, and the likelihood of continuity in different types of IPV perpetration across the waves (Table 4). The results showed that general offending was significantly associated with the likelihood of continuity in various forms of IPV perpetration. The effects of general offending became slightly smaller when experience of family violence and low self-control were added to the models, but all effects remained (marginally) significant (only full models are shown in Table 4). Furthermore, respondents with a higher educational level and those who had experienced family violence showed an increased likelihood of continuity in any IPV and psychological IPV perpetration. Dutch-Moroccan respondents were at increased risk of showing continuity in sexual coercion across the waves. In addition, males were more likely than females to show continuity in sexually coercive IPV, and less likely than females to show continuity in psychological IPV, although both effects were marginally significant. Finally, young adults with lower levels of self-control were more likely to show continuity in sexual coercion and physical IPV, albeit the latter effect was marginally significant.

**Table 4.** The Association between General Offending, Demographic and Individual Characteristics, and Continuity in IPV Perpetration over the Waves (*N* = 352).

	Model 5: Continuity in any IPV		Model 6: Continuity in psychological IPV		Model 7: Continuity in physical IPV		Model 8: Continuity in sexual coercion	
	B	*SE*	B	*SE*	B	*SE*	B	*SE*
General offending	0.08*	0.04	0.07^†^	0.04	0.08**	0.03	0.06*	0.03
Age	−0.15	0.11	−0.13	0.10	−0.05	0.11	−0.17	0.19
Gender	−0.37	0.26	−0.47^†^	0.26	−0.24	0.28	0.89^†^	0.49
Dutch-Antillean	0.48	0.36	0.18	0.35	0.50	0.34	1.05	0.66
Dutch-Moroccan	−0.30	0.31	−0.48	0.31	0.22	0.33	1.67**	0.55
Medium education level	0.47	0.40	0.76†	0.39	0.36	0.46	−0.62	0.71
High education level	0.85*	0.41	1.00*	0.40	0.55	0.47	0.20	0.75
Family violence	1.02**	0.39	1.21**	0.39	0.29	0.32	0.19	0.53
Low self-control	0.46	0.43	0.32	0.42	0.87^†^	0.47	1.83*	0.78
Constant	2.06	2.22	1.96	2.19	−2.87	2.38	−4.96	4.17

† *p* < .10. *p < .05. **p < .01.

Taken together, the results not only point to an important association between general offending and IPV perpetration in each wave, but also indicate that involvement in more diverse general criminal behavior is associated with an increased likelihood of continuity in IPV perpetration across the waves. This suggests that young adults who show diverse general offending may be the most serious IPV perpetrators and at the highest risk of extending their abusive behaviors beyond young adulthood.

## Discussion

Using data from a longitudinal study of young adults from the Netherlands, this study examined the association between general offending and IPV perpetration during early adulthood. Results from this Dutch study speak to the generalization of findings from similar research carried out predominantly in the US.

Like studies from other countries (e.g., Johnson et al., 2015; Kim et al., 2008; Shortt et al., 2012), the findings of this study indicate that IPV perpetration in young adulthood is not uncommon, with over three-quarter of young adults reporting psychological IPV, and over 40% engaging in physical IPV perpetration in wave 1. Sexual coercion was least prevalent across the waves. Prevalence and frequency of IPV perpetration generally decreased over the waves. However, a considerable proportion of young adults showed continuity in IPV perpetration over the waves, particularly regarding psychological IPV. Furthermore, approximately one in three young adults reported involvement in general offending in each wave, but the average number of different offenses reported was relatively low.

Using random effects models, we examined the association between general offending, demographic and individual factors, and IPV perpetration. Across all models, general offending was significantly associated with different types of IPV perpetration, indicating that young adults engaged in higher rates of IPV perpetration in those waves in which they reported more diverse offending. While the effects became slightly smaller when demographic and individual factors were added to the models, all remained significant. Moreover, results from logistic regression analyses demonstrated that general offending was associated with a likelihood of continuity in IPV perpetration.

Taken together, findings from this study clearly indicate that it is useful to see IPV perpetration as part of a broader criminal career. Our findings are consistent with research conducted in other industrialized countries that found a significant association between general antisocial or criminal behavior and IPV perpetration (e.g., Capaldi & Clark, 1998; Ehrensaft et al., 2003; Herrenkohl et al., 2007; Kim & Capaldi, 2004; Magdol et al., 1998; Moffitt et al., 2002; Novak & Furman, 2016; Piquero et al., 2014; Verbruggen et al., 2020; Woodward et al., 2002). Our study provides additional support for general crime and violence theories that assume different manifestations of antisocial behavior are related to each other due to their shared underlying risk factors (Fagan & Wexler, 1987; Felson & Lane, 2010; Gottfredson & Hirschi, 1990; Moffitt, 1993).

Another consideration raised by our research is the role of two key background risk factors, which according to general theories of crime and violence are thought to mediate the association between general crime and IPV. Firstly, in all random effects models, those with lower levels of self-control (or higher levels of “antisocial propensity”) reported significantly more IPV perpetration (Payne et al., 2010; Sellers, 1999). Moreover, low self-control was related to an increased likelihood of continuity in physical IPV and sexual coercion in relationships. These results support Gottfredson and Hirschi’s (1990) view that various forms of antisocial behavior stem from a stable underlying criminal propensity. However, general offending was still significantly correlated with IPV perpetration, even after we considered low self-control. This suggests that an underlying antisocial propensity such as a low level of self-control only partially explains the association between general offending and IPV, and that other factors are also important in explaining IPV, including, as hypothesized by integrated models of IPV, dynamic and/or proximal factors such as substance use and psychopathology (Bell & Naugle, 2008; Capaldi & Kim, 2007; Capaldi et al., 2005; DeMaris et al., 2003), as well as meso and macro factors such as socioeconomic, neighborhood, and cultural characteristics (Beyer et al., 2015; Sijtsema et al., 2020).

Second, witnessing and/or experiencing family violence in childhood was found to be a key risk factor for IPV perpetration. In this study, witnessing and/or experiencing family violence in childhood was significantly related to levels of physical IPV perpetration, as well as to psychological IPV and the likelihood of continuity in psychological violence, also when general offending was taken into account. These findings support previous research on experiencing child abuse and family violence as an important factor in explaining different types of IPV perpetration (Gómez, 2011; Sunday et al., 2011) and the “cycle of violence” hypothesis (Mihalic & Elliott, 1997; Widom, 1989).

Regarding demographic characteristics, in accordance with prior research using the Conflict Tactics Scale to measure IPV (Archer, 2000; Straus, 2004), no gender differences were found in psychological and physical IPV perpetration. However, males reported higher rates of sexually coercive IPV than females. Second, Dutch-Antillean and Dutch-Moroccan respondents reported more acts of sexual coercion than Dutch respondents, while Dutch-Moroccan young adults were also more likely to show continuity in sexual coercion. US-based research also found that ethnic minorities were at increased risk of IPV perpetration, although this association was partly explained by socioeconomic inequalities (Benson et al., 2004). Third, a higher educational level was associated with increased rates of psychological and physical IPV perpetration, and with continuity in psychological violence. It is possible that the higher educated young adults in this study had a better awareness and understanding of partner violence, due to school, university and media campaigns, and therefore recognize and report more IPV behaviors (Fellmeth et al., 2013; Whitaker et al., 2013). Given that other research has shown lower educational attainment to be a risk factor for IPV (Capaldi et al., 2012), the available evidence remains inconclusive. Finally, the oldest individuals in our sample reported less IPV perpetration, pointing to a decrease in IPV with age, consistent with other studies (Johnson et al., 2015; Kim et al., 2008; Shortt et al., 2012).

The key finding from our study is that general offending, especially when it manifests as involvement in more diverse offending, is related to both the perpetration of a higher volume of IPV offenses and the likelihood of continuity in IPV over time. By demonstrating an important overlap between general criminal behavior and IPV perpetration among young adults from the Netherlands, this study adds to a growing body of scholarship, thereby pointing to the generalizability of these findings. However, this study has the following limitations.

First, it is unclear to what extent the results are representative of all Dutch young adults. The TransAM study focused on young adults from the Netherlands’ largest city. Moreover, due to the study’s focus on criminal behavior, those who have had police contact prior to age 17, as well as Dutch-Moroccan young adults and Dutch-Antillean young adults, were oversampled. Available information suggests that the study was successful in obtaining a representative sample of urban young adults, although, in line with the study aims, 19.2% of the final sample had a police record before age 17, compared to 10.0% of the study’s population of young adults (Hill et al., 2016). Furthermore, given the current study’s focus on IPV perpetration, we examined a selective subsample of respondents who had been in a relationship in at least two out of the four waves. To illustrate, this subsample consisted of a higher proportion of females (60.8%) compared to the original sample (54%–58% across the waves) (Hill et al., 2016), and included respondents were older and relatively well educated. Taken together, these issues may impact the generalizability of the findings.

Second, general offending and IPV perpetration may have been under-reported in this study, especially among Moroccan young adults (Stevens et al., 2003; Van der Laan & Blom, 2011). Third, the CTS2 was used in this study to measure IPV perpetration, but there are debates over the extent to which this instrument can accurately measure IPV (Dobash et al., 1992). Moreover, some have argued that the CTS2 disproportionately captures “situational couple violence,” which is a relatively minor and more often mutual form of IPV perpetration (Johnson, 2008). This type of violence is often contrasted with another form of IPV labeled “intimate terrorism,” which is characterized by ongoing, systematic violence and coercive controlling behavior (Johnson, 2008; Stark, 2007). The absence of gender differences in most forms of IPV perpetration in this study, and the overlap between perpetration and victimization,^6^ suggests that the CTS2 captured many instances of situational couple violence. Nevertheless, the findings also show that the CTS2 can effectively measure both continuity in IPV perpetration over time, and the simultaneous use of different types of IPV perpetration. This suggests that our study has also captured more serious and persistent IPV perpetrators (i.e., those whose profile of abusive behaviors can be described as intimate terrorism).

### Directions for Future Research and Implications for Interventions

The current study contributes to the burgeoning area of research on general offending and IPV perpetration by demonstrating that more diverse offending is associated with higher rates of IPV perpetration as well as the likelihood of continuity in IPV over time. Future research could build upon these findings in two important ways.

First, the results of this study point to the importance of a history of experiencing family violence and low levels of self-control, in addition to general offending, when explaining IPV perpetration. Future longitudinal research should aim to also consider how other potentially relevant factors proposed in extant theoretical models of IPV, including individual characteristics such as psychopathology (Henrichs et al., 2015; Holtzworth-Munroe & Stuart, 1994), and meso and macro factors such as socioeconomic, neighborhood and cultural characteristics (Beyer et al., 2015; Sijtsema et al., 2020) impact the relationship between general offending and IPV perpetration in order to further our understanding of IPV development.

Second, this study indicated, like previous research, that IPV perpetration tends to decrease with age. Although there is a growing body of research examining the development of IPV over time using different samples, including adolescents (Goncy et al., 2018; O’Leary & Slep, 2003), young adults (Capaldi et al., 2003; Kim et al., 2008; Saint-Eloi Cadely et al., 2017) and married couples (Aldarondo, 1996; Caetano et al., 2005; Jasinski, 2001; Lorber & O’Leary, 2004; Woffordt et al., 1994), little is known about factors that promote desistance from IPV perpetration (Walker et al., 2013). Therefore, we recommend that future longitudinal research seeks to identify the contextual and time-dependent factors related to desistance from IPV perpetration, including those found to be associated with desistance from general crime, such as employment (Sampson & Laub, 1993).

To conclude, young adults in this study perpetrated higher levels of IPV at times when they were also involved in general offending. This key finding yields important implications for prevention and intervention. In addition to primary prevention initiatives designed to prevent antisocial, violent, and abusive behaviors among youths generally, this study indicates that secondary prevention efforts targeted at high-risk youths may be especially useful for reducing IPV (Capaldi & Langhinrichsen-Rohling, 2012; Ehrensaft, 2008). Young adults who are involved in more diverse offending behavior tend to show higher rates of different types of IPV perpetration. Identifying these criminally active young adults when they come into contact with criminal justice agencies due to their general offending behavior could provide an avenue to prevent and reduce IPV perpetration, which is less likely to become known to the authorities (FRA, 2014). Furthermore, even though involvement in IPV perpetration decreases throughout early adulthood for most young people, it is crucial to focus intervention efforts on the smaller group of young adults with a more extensive criminal career, who show continuity in their abusive behavior, and are therefore at heightened risk of extending their IPV perpetration beyond young adulthood (Verbruggen et al., 2019). Preventing persistence in IPV perpetration does not only reduce further victimization of partners, it also decreases the risk of children’s exposure to violence, thereby lowering the chances of intergenerational transmission of violence (Sijtsema et al., 2020; Widom, 1989).

## Notes

1. This subsample of 352 respondents were not significantly different from those who have been in a relationship once during the study period (*N* = 203) with regard to gender, ethnicity, age at wave 1, family violence in childhood, level of self-control, any involvement in general offending in the different waves, and the average number of different offenses reported in the different waves. However, there was a significant difference in terms of educational level, as a smaller proportion of respondents with a low educational level, compared to a medium or high level of education, were included in the subsample (χ²(2, 555) = 6.92, *p* < .05). When comparing the subsample of respondents who reported being in a relationship in at least two out of the four waves (*N* = 352) to those who were only in one wave, or never, in a relationship (*N* = 618), there were no significant differences between these two groups regarding family violence in childhood, level of self-control, any involvement in general offending in the different waves, and the average number of different offenses reported in the different waves. However, respondents who were in a relationship in at least two waves were older (*M* = 20.12, *SD* = 1.31) than those excluded from the subsample (*M* = 19.86, *SD* = 1.36) (*t*(968) =  −2.87, *p* < .01). There was also a significantly higher proportion of females (40.6% of all females that participated in wave 1) than males (31.2% of all males that participated in wave 1) included in the subsample (χ²(1, 970) = 9.31, *p* < .01). Furthermore, a lower proportion of Dutch-Moroccan participants (24.0%) had been in a relationship in at least two waves, compared to those from Dutch (44.3%) or Antillean backgrounds (42.5%) (χ²(2, 970) = 38.87, *p* < .01). Moreover, respondents with a low educational level were underrepresented in the subsample (χ²(2, 970) =  9.44, *p* < .01).

2. As part of the self-reported offending questionnaire, respondents were asked what the relationship was between them and the victim. Nine respondents who had committed a violent offense reported that the victim of that offense was an (ex)partner. To avoid overlap between the self-report general offending measure and the IPV perpetration variables, we have excluded the relevant violent offenses from the general offending measure for these nine respondents.

3. Two respondents had another ethnic background, and were coded as Dutch.

4. There were no significant gender differences in the occurrence of most forms of IPV perpetration across the waves. However, a higher proportion of males (27.9%) than females (18.8%) reported physical IPV in wave 3, but this difference was only marginally significant (χ²(1, 269) =  3.05, *p* = .08). Moreover, males were significantly more likely than females to report sexual coercion in wave 1 (19.6% of males, 4.5% of females; χ²(1, 283) = 16.46, *p* < .01) and wave 3 (20.2% of males, 5.5% of females; χ²(1, 269) = 13.98, *p* < .01).

5. These conclusions hold when different operationalizations of measures (i.e., dichotomous measures of IPV perpetration, the original scale measure of IPV, or a frequency measure rather than a variety measure for self-report general offending) had been used.

6. There was a significant association between the different types of IPV perpetration and victimization in all waves: any IPV wave 1 (χ²(1, 283) = 161.95, *p* < .01), wave 2 (χ²(1, 275) = 168.47, *p* < .01), wave 3 (χ²(1, 269) = 182.54, *p* < .01), wave 4 (χ²(1, 245) = 163.42, *p* < .01). Psychological IPV wave 1 (χ²(1, 283) = 151.62, *p* < .01), wave 2 (χ²(1, 275) = 177.05, *p* < .01), wave 3 (χ²(1, 269) = 179.06, *p* < .01), wave 4 (χ²(1, 245) = 171.01, *p* < .01). Physical IPV wave 1 (χ²(1, 283) = 146.91, *p* < .01), wave 2 (χ²(1, 275) = 194.06, *p* < .01), wave 3 (χ²(1, 269) = 177.47, *p* < .01), wave 4 (χ²(1, 245) = 153.91, *p* < .01). Sexual coercion wave 1 (χ²(1, 283) = 121.13, *p* < .01), wave 2 (χ²(1, 275) = 112.73, *p* < .01), wave 3 (χ²(1, 269) = 128.58, *p* < .01), wave 4 (χ²(1, 245) = 113.99, *p* < .01).
